# Spontaneous intraperitoneal rupture of hepatic hydatid cyst in a cirrhotic patient: A diagnostic and therapeutic challenge – A case report

**DOI:** 10.1016/j.ijscr.2025.111552

**Published:** 2025-06-21

**Authors:** Ben Hassine Basma, Rabti Souphia, Ferjaoui Wael, Ben Marzouk Saoussen, Lahouar Omar, Khalifa Mohamed Bechir

**Affiliations:** a General Surgery Department, Military Hospital of Tunis, Mont Fleury-1008, Tunis, Tunisia; b Faculty of Medicine of Tunis, 15, Djebel Lakhdhar Street – 1007 Bab Saadoun, Tunis, Tunisia; c Faculty de Medicine Ibn El Jazzar of Sousse, Mohamed Karoui Street - 4002, Sousse, Tunisia; d Faculty of Medicine of Monastir, Avenue Avicenne- 5000, Monastir, Tunisia

**Keywords:** Hydatid disease, Echinococcus, Hydatid cyst, Intraperitoneal rupture, Peritonitis, Cirrhosis

## Abstract

**Introduction:**

Hydatid disease, caused by Echinococcus granulosus, is a parasitic condition typically considered benign. However, the rupture of a hepatic hydatid cyst into the abdominal cavity is a rare and potentially fatal complication. This complication is particularly complex in cirrhotic patients, posing significant diagnostic and therapeutic challenges.

**Case report:**

We present a case of a 64-year-old woman with a history of poorly managed post-viral cirrhosis due to hepatitis C who presented with abdominal pain lasting for ten days. A computed tomography (CT) scan revealed the rupture of a hydatid cyst in the segment VIII of the liver, associated with a peritoneal reaction. An emergency exploratory laparotomy was performed, confirming the rupture and allowing for the resection of the protruding cyst dome, followed by peritoneal lavage and drainage. No recurrence was observed during a six-month follow-up.

**Discussion:**

Hydatid disease is endemic in Tunisia and poses a significant public health concern. Although rare (1 to 16 %), intraperitoneal rupture is a severe complication. CT is a key diagnostic tool, offering high sensitivity, especially for differential diagnoses. The management of this condition necessitates urgent surgical intervention, followed by medical treatment with albendazole to prevent recurrence.

**Conclusion:**

This case underscores the rarity of intraperitoneal rupture of a hydatid cyst and emphasizes the importance of considering this complication in the diagnosis of abdominal emergencies in endemic regions, as delayed diagnosis is associated with a particularly poor prognosis.

## 1 Introduction

Hydatid disease, a parasitic zoonosis caused by Echinococcus granulosus, represents a significant public health issue in endemic regions, particularly in Tunisia [1,2]. Humans, acting as accidental intermediate hosts, contract the infection through the fecal-oral route [3].

This pathology primarily affects the liver, which serves as the main barrier against parasite dissemination and remains the most frequently affected organ (50 % to 77 % of cases) [3,4]. Intraperitoneal rupture of the hepatic cyst, though rare (with an incidence of 1 % to 16 %), is a potentially fatal event that requires urgent, multidisciplinary management [5].

This situation is even more complex in cirrhotic patients, where early diagnosis and appropriate therapeutic intervention are essential to prevent a fatal outcome.

This work has been reported in line with the SCARE criteria [6].

## 2 Case presentation

This work has been reported in line with the SCARE criteria [6].

We present a case study of a 64-year-old woman from a rural area in Tunisia. She had a history of poorly managed post-viral hepatitis C cirrhosis. She presented to the emergency department with abdominal pain localized to the right hypochondrium, accompanied by nausea and vomiting. The symptoms had gradually worsened over the past ten days. The patient reported no history of trauma.

On clinical examination, vital signs were stable. The physical examination revealed a fever of 38.8 °C, abdominal distension with dullness, and tenderness in the right upper quadrant on palpation.

Laboratory tests showed leukocytosis at 14,000/μL, eosinophilia at 1.8 G/L, and an elevated CRP level of 82 mg/L. Liver function tests revealed mild elevation in transaminases (AST: 64 U/L, ALT: 72 U/L), while bilirubin and alkaline phosphatase levels remained within normal limits. The analysis of the ascitic fluid, which was slightly cloudy, showed an exudative fluid with a predominance of lymphocytes, suggesting a superinfection.

**Fig. 1:**
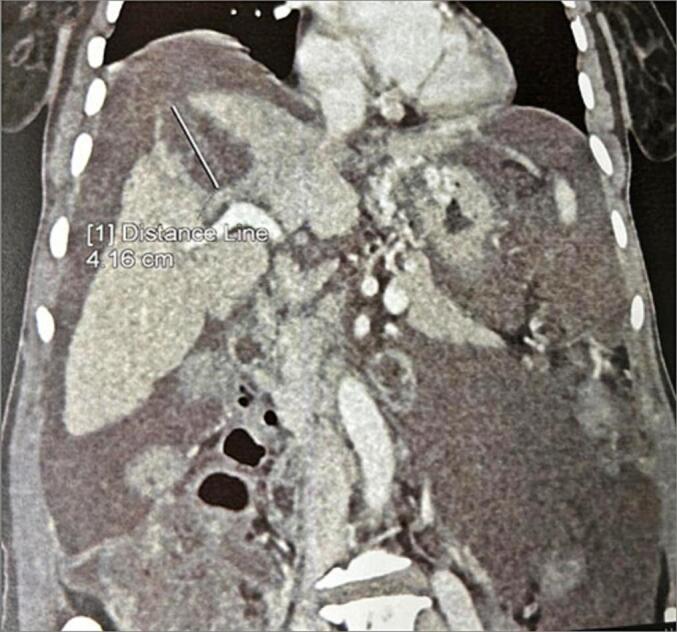
Abdominal CT scan showing a dysmorphic liver with a ruptured hydatid cyst in segment VIII (white arrow) and associated peritoneal effusion (asterisk).

CT imaging of the abdomen revealed a dysmorphic liver containing a 4 cm hydatid cyst in the VIII segment, with a discontinuous wall, associated with pericystic structures and diffuse intraperitoneal effusion, suggesting acute peritonitis secondary to spontaneous rupture of a hepatic hydatid cyst (Fig. 1).

**Fig. 2:**
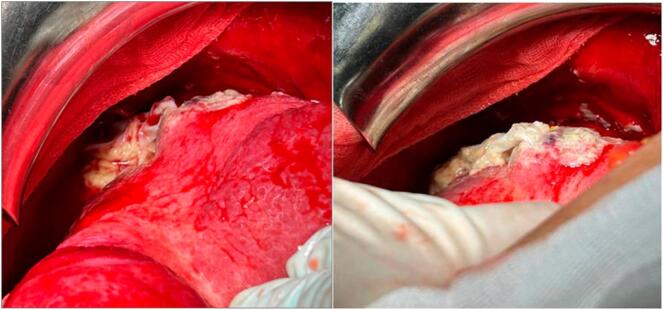
Intraoperative findings showing the ruptured hydatid cyst (arrow) at the hepatic dome and free daughter cysts in the peritoneal cavity (arrowheads).

Emergency exploratory laparotomy confirmed the rupture of a 5 cm hydatid cyst located at the hepatic dome (Fig. 2). The removal of daughter cysts was followed by extensive lavage of the cystic cavity with hypertonic saline solution to inactivate any remaining protoscolices. Resection of the prominent dome was also carried out. Two drains were placed subhepatically and in the interhepato-diaphragmatic space.

The postoperative evolution was favorable, and the patient was discharged on the sixth day with a prescription for albendazole to prevent recurrence. A six-month follow-up revealed no signs of recurrence.

## 3 Discussion

Echinococcus granulosus is a cosmopolitan parasite, particularly prevalent in sheep farming regions, including North Africa, Asia, South America, and the Mediterranean region [7]. Hydatid disease is more common in rural areas where conditions facilitate its transmission. In Tunisia, this pathology remains frequent and endemic [8].

Hydatid disease can affect any organ in the human body, but it most commonly involves the liver, which is affected in 50 % to 77 % of cases. The lungs are involved in 15 % to 47 % of cases, while other sites, such as the spleen (0.5 % to 8 %) and kidneys (2 % to 4 %), are less frequently affected [9]. Hepatic involvement predominantly affects the right lobe in 80 % of cases, and cysts may be either single or multiple, with the latter accounting for 20 % to 40 % of cases [10,11].

The coexistence of hydatid cyst with liver cirrhosis is uncommon, with limited literature available on this specific condition. A review by Bosch et al. demonstrated that in cirrhotic patients, the altered hepatic architecture can mask typical imaging features of hydatid cysts, potentially delaying diagnosis [17]. Additionally, the compromised immune status of cirrhotic patients may predispose them to a higher risk of cyst complications, including rupture and secondary infection.

In female cirrhotic patients specifically, hormonal factors may influence the course of the disease. Azizi et al. reported that female patients with Child-Pugh B cirrhosis have a 1.8-fold increased risk of hydatid cyst complications compared to males with similar liver function status [18]. Our case aligns with these observations, as our patient exhibited symptoms that initially appeared to be related to her underlying cirrhosis, potentially masking the evolving hydatid cyst rupture.

In the majority of cases, hepatic hydatid cysts are asymptomatic and are discovered during routine radiological examinations. The most common complications include infection and rupture, which can occur in the biliary tract, peritoneum, or thorax [5,9].

In our case, the patient's presentation with progressive abdominal pain, fever, and ascites initially suggested a complication of her underlying cirrhosis. However, the presence of eosinophilia (1.8 G/L) provided an important diagnostic clue, as this finding is uncommon in uncomplicated cirrhosis but typical in parasitic infections. The ascitic fluid analysis showing an exudative pattern with lymphocyte predominance further supported an inflammatory or infectious process beyond simple cirrhotic ascites.

Intraperitoneal rupture, although rare (1 to 16 %), poses a significant challenge in terms of diagnosis and treatment [1]. It can be spontaneous, typically due to an increase in intra-cystic pressure, as in our case, or traumatic, occurring during a perioperative procedure or abdominal trauma [4,12].

There are two distinct forms of hydatid cyst rupture into the peritoneal cavity. The minor fissure, the most common type, usually occurs secondary to mild trauma and may cause a cutaneous allergic reaction in 16 % to 25 % of cases [3]. The released hydatid fluid may either become encapsulated or progress to diffuse peritoneal hydatidosis [8]. In contrast, massive rupture, often resulting from severe abdominal trauma, can lead to an acute, life-threatening condition with an estimated risk of fatal anaphylactic shock of 1.4 % [9].

A thorough clinical evaluation is essential to prevent diagnostic delays, as symptoms are often nonspecific and may mimic other acute abdominal emergencies [13].

A diagnostic approach combining clinical examination, biological tests, and imaging is crucial for confirming rupture and guiding therapeutic management. A complementary biological assessment may reveal leukocytosis and elevated inflammatory markers, suggesting an underlying infectious or inflammatory process [14].

Imaging, particularly computed tomography (CT), remains the reference examination, especially in hemodynamically stable patients, due to its high sensitivity (100 %) [9]. CT allows visualization of characteristic signs, such as a reduction in cyst size, a discontinuous wall, as well as the presence of fluid or daughter cysts in the peritoneal cavity. Although less sensitive (85 %), ultrasound remains a useful non-invasive method for detecting intra-abdominal effusion and the presence of a floating membrane [4,15].

The patient's management should begin immediately in the emergency department, with close monitoring [3].

Surgical treatment is based on key principles, including abundant irrigation with physiological saline combined with scolocidal solutions to prevent peritoneal recurrences [11,14]. In our case, we opted for resection of the protruding dome, which is the most frequently employed technique in emergency situations (83 % of cases).

The choice of dome resection rather than radical surgery (pericystectomy or hepatic resection) was based on several considerations unique to our patient's condition. In cirrhotic patients, major hepatic resections carry significantly higher morbidity and mortality rates due to impaired liver function and potential coagulopathy [19]. Dome resection offers several advantages in this context:

1. Preservation of functional liver parenchyma, which is critical in cirrhotic patients
2. Reduced operative time, minimizing anesthesia-related complications
3. Lower risk of bleeding, a particularly important consideration in patients with portal hypertension and potential coagulopathy

Recent studies support this approach in cirrhotic patients. Manterola et al. demonstrated that conservative surgical techniques like dome resection have comparable long-term outcomes to radical approaches in terms of recurrence rates (7.2 % vs. 6.8 % at 5 years) but with significantly lower perioperative complications in patients with compromised liver function [20].

To prevent recurrences, adjunctive medical treatment with albendazole, along with liver function monitoring, is essential [11]. Our patient received a standard regimen of albendazole (10–15 mg/kg/day) for three months with close monitoring of liver enzymes, which is particularly important given her underlying cirrhosis.

## 4 Conclusion

Intraperitoneal rupture of hydatid cysts is a severe complication of cystic echinococcosis that can lead to significant hemodynamic instability and potentially fatal allergic reactions. Therefore, it is crucial to consider this diagnosis in patients presenting with abdominal pain, particularly in endemic areas.

This condition poses a significant challenge for emergency physicians, radiologists, and surgeons. The management of this complication is primarily surgical, supplemented by postoperative medical treatment to prevent recurrence and ensure effective risk management. The presence of underlying cirrhosis further complicates both diagnosis and treatment, requiring a tailored approach to optimize outcomes.

## Author contribution

Rabti Souphia and Ben Marzouk Sawssen contributed to manuscript writing and editing, and data collection; Wael Farjaoui, Ben Hassine Basma contributed to data analysis; Med Bachir Khalifa contributed to conceptualization and supervision; All authors have read and approved the final manuscript.

## Patient consent

Written informed consent was obtained from the patient for the publication of this case report and its accompanying images. A copy of the written consent is available for the Editor-in-Chief of this journal to review upon request.

## Ethical approval

Ethical approval is not applicable/waived at our institution. Due to the specific nature of case reports, which involve detailed descriptions of observations and interventions that have already been conducted on patients, as opposed to prospective studies involving planned interventions, our institution does not require formal ethical approval for such cases. We recognize the importance of ethics in medical research and are fully committed to upholding ethical standards in our medical and research practices.

## Guarantor

Rabti Souphia.

## Research registration number

N/A.

## Funding

This research did not receive funding from any specific grant provided by public, commercial, or not-for-profit organizations.

## Conflict of interest statement

No conflicts of interest.
